# Designing anisotropic porous bone scaffolds using a self-learning convolutional neural network model

**DOI:** 10.3389/fbioe.2022.973275

**Published:** 2022-09-27

**Authors:** Yongtao Lu, Tingxiang Gong, Zhuoyue Yang, Hanxing Zhu, Yadong Liu, Chengwei Wu

**Affiliations:** ^1^ Department of Engineering Mechanics, Dalian University of Technology, Dalian, China; ^2^ DUT-BSU Joint Institute, Dalian University of Technology, Dalian, China; ^3^ Xi’an Aerospace Propulsion Institute, Xi’an, China; ^4^ School of Engineering, Cardiff University, Cardiff, United Kingdom; ^5^ Department of Orthopedics, Dalian Municipal Central Hospital Affiliated of Dalian University of Technology, Dalian, China; ^6^ State Key Laboratory of Structural Analysis for Industrial Equipment, Dalian University of Technology, Dalian, China

**Keywords:** scafffold design, convolutional neural network, anisotropic property, bone tissue, finite element modeling

## Abstract

The design of bionic bone scaffolds to mimic the behaviors of native bone tissue is crucial in clinical application, but such design is very challenging due to the complex behaviors of native bone tissues. In the present study, bionic bone scaffolds with the anisotropic mechanical properties similar to those of native bone tissues were successfully designed using a novel self-learning convolutional neural network (CNN) framework. The anisotropic mechanical property of bone was first calculated from the CT images of bone tissues. The CNN model constructed was trained and validated using the predictions from the heterogonous finite element (FE) models. The CNN model was then used to design the scaffold with the elasticity matrix matched to that of the replaced bone tissues. For the comparison, the bone scaffold was also designed using the conventional method. The results showed that the mechanical properties of scaffolds designed using the CNN model are closer to those of native bone tissues. In conclusion, the self-learning CNN framework can be used to design the anisotropic bone scaffolds and has a great potential in the clinical application.

## 1 Introduction

Every year, millions of bone replacement surgeries have to be performed worldwide to fix the large bone defects (Dang et al., 2018). In these surgeries, the autograft and allograft are the techniques widely used in the clinic, but in autograft there is the issue associated with the lack of bone supply and in allograft there are issues, such as the disease transmission (Finkemeier, 2002; Laurencin et al., 2006). Because of these, the bone scaffold has emerged as a promising method for fixing large bone defects. However, there are still many issues to be solved, such as the stress shielding. The structural design is one of the main approaches to solve these challenges but it is still in its early stage and requires extensive research.

In the structural design of bone scaffolds, the design target is to have a bionic scaffold which can mimic the behaviors of the defected native bone tissues in all aspects including the geometrical features, the mechanical and biological functions, etc. However, it should be noted that geometrically the native bone possesses irregular shapes and mechanically the bone is anisotropic in different scales, which poses a big challenge in the bionic design of bone scaffolds. In the past, many efforts have been made to achieve the bionic design of bone scaffolds. For example, the microstructure of bone scaffolds has been evolved from the periodic regular lattices to the triply periodic minimal surface (TPMS) based structures and further to the irregular, non-periodic structures (Vijayavenkataraman et al., 2018; Huo et al., 2021). The periodic regular lattices (e.g., the cube, the hexagon) are widely used in the early stage of the design of bone scaffold (Bucklen et al., 2008). The TPMS based scaffold is one of the main types of structures widely used nowadays because of the bionic features of the TPMS, such as a mean curvature of zero. Most recently, the focus of the scaffold design has been put on the anisotropic behaviors of TPMS scaffolds (Ataee et al., 2018; Bonatti and Mohr, 2019; Kang et al., 2020; Peng et al., 2021). For example, Peng et al. (2021) has managed to increase the range of scaffold anisotropy by modifying the geometrical parameters of Gyroid cellular structure. In addition, the techniques such as grading and hybrid design, etc. are used to design the scaffolds with anisotropic mechanical properties (Liu et al., 2018; Al-Ketan et al., 2019; Chen et al., 2019). However, because the TPMS based scaffolds are based on the periodic units and additionally the number of design variables in the TPMS scaffolds is limited, the design space for the anisotropic mechanical properties of the TPMS scaffolds is limited. Designing the irregular and non-periodic bone scaffolds using some advanced mathematical algorithms (e.g., the Voronoi algorithm) (Gómez et al., 2016; Wang et al., 2018) is one of the strategies to achieve a larger design space for the anisotropic mechanical properties, but high complexities are involved in the advanced mathematical algorithms which hinders its development and application. Therefore, a novel and efficient approach for designing the scaffolds with controllable anisotropy is still highly needed.

In recent years, the machine learning has been evolved as a novel and fast-growing technique, which has been successfully applied in many fields, e.g., the accurate prediction of musculoskeletal force (Rane et al., 2019), the automatic tracking of joint kinematics (Burton et al., 2021), etc. In the design of porous materials, the machine learning based technique has also been widely explored in the recent years. For example, Zheng et al. (2021) has managed the inverse design of auxetic metamaterials using deep learning; Gu et al. (2018) has managed the design of bioinspired hierarchical composite using machine learning; a deep-learning based model was proposed by Tan et al. (2020) for the efficient design of microstructural materials. The advantage of the machine learning technique is that once the machine learning model is well trained and validated, it can serve as an efficient surrogate model for generating the real-time outputs from new inputs. Additionally, the machine learning based technique is able to deal with structural design involving a large number of design variables, which is extremely crucial in the design of porous scaffolds, because the design space for the scaffold anisotropy can be easily expanded by adding more design variables. Therefore, the machine learning technique has the great potential in designing the fully bionic bone scaffolds. Nevertheless, to the best of our knowledge, the design of bone scaffolds with anisotropic mechanical properties using the machine learning technique has not been fully elaborated. The aim of the present study was to design bionic bone scaffolds with the mechanical properties similar to those of native bone tissues using the emerging machine learning technique.

This paper is organized in the following scheme. The calculation of the elasticity matrix of native bone tissue and the details on the self-learning convolutional neural network based design framework are illustrated in Section 2. In Section 3, the performance of the developed framework is demonstrated using a two-dimensional (2D) bone sample. In Section 4, the predictive accuracy of the machine learning based model and the design results are discussed and conclusions are drawn in the end.

## 2 Materials and methods

### 2.1 Calculation of the elasticity matrices for the native bone and bone scaffold

**FIGURE 1:**
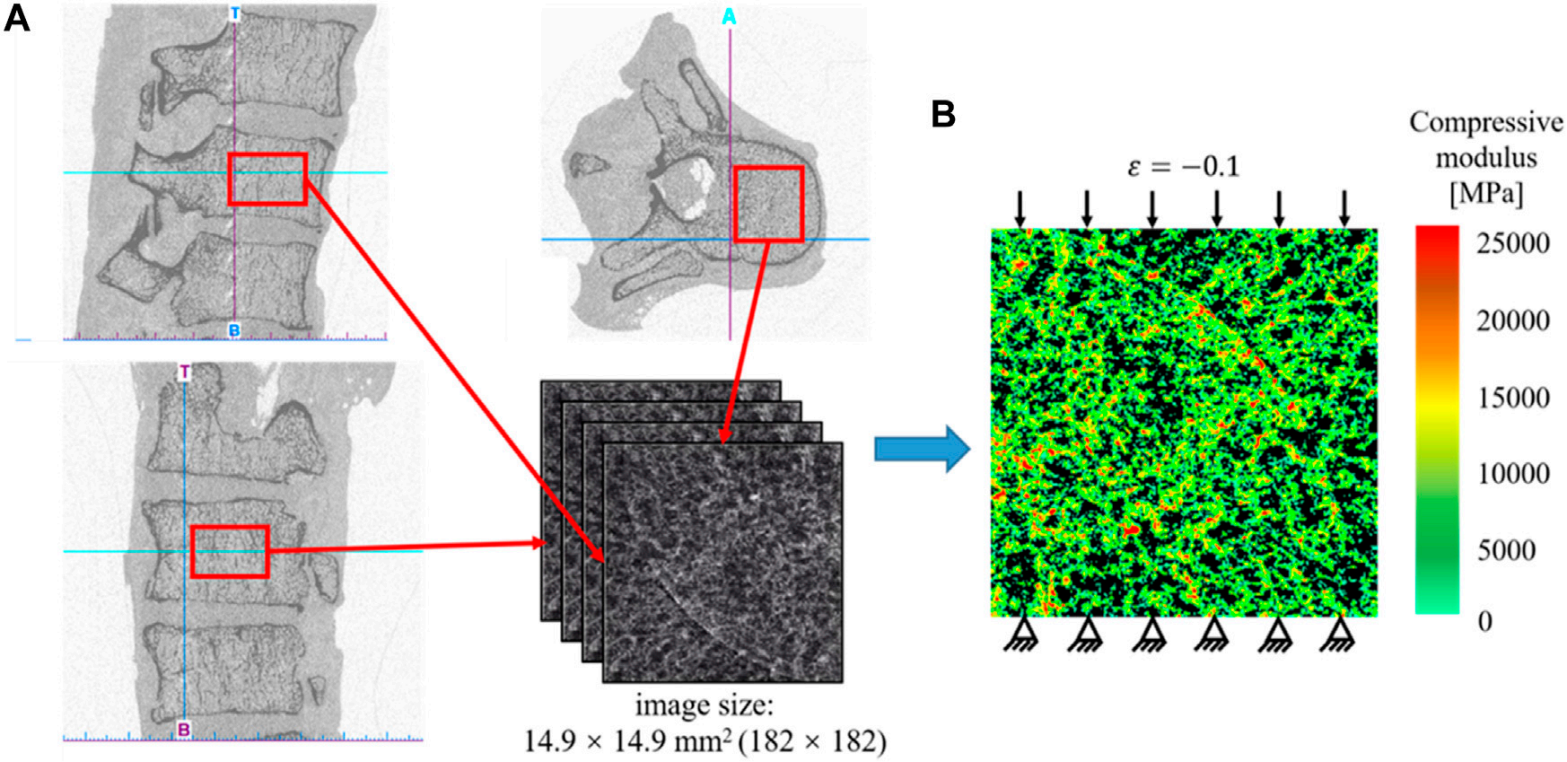
**(A)** Extraction of the CT data of the human cancellous bone. **(B)** Establishment of the heterogeneous finite element model for calculating the effective elastic mechanical modulus of bone tissue.

In the present study, a 2D bone example is presented to demonstrate the application of the machine learning based method in the design of the anisotropic porous bone scaffold. The anisotropic bone scaffold to be designed is to replace the defected bone tissues and an ideal scaffold should possess the mechanical properties similar to those of the replaced bone tissue. Therefore, the first step in the scaffold design is to work out the mechanical properties of bone tissue, which are calculated from the CT images of native human bone tissue. The CT images used were acquired in the previous studies (Lu et al., 2015). Briefly, thirty-five cadavers were harvested from the female patients with a mean age of 81.3 ± 7.2 year-old (range: 65 to 90 year-old). The spinal segments of T11/T12/L1 were dissected and the specimens were scanned in the frozen state using the HR-pQCT scanner (XtremeCT, Scanco Medical AG, Bruettisellen, Switzerland) operated at 59.4 kV, 900.0 μAs with an image voxel size of 82.0 × 82.0 × 82.0 μm^3^. In the present study, only the cancellous fraction was used and thus the volumes of interest covering only the cancellous bone were cropped out from the CT images of the spinal segments (Figure 1A).

Because the native bone tissue exhibits the anisotropic mechanical properties, the homogenized elasticity matrix of the bone was calculated to describe the anisotropic mechanical properties of the native bone tissue, which were calculated from the finite element (FE) analysis as follows (Figure 1). First, the heterogeneous FE models of the cancellous fraction were generated using the standard method previously developed (Lu et al., 2019a). Briefly, the cancellous fraction (Figure 1A) with the dimension of 14.9 × 14.9 mm^2^ was cropped out from the HR-pQCT images of human vertebral body. The grayscale image was first smoothed using a Gaussian filter (sigma = 1.2, support = 2.0) to reduce the influence of image noise. Then, the grayscale values were converted to vBMD values based on the linear calibration equation provided by the HR-pQCT scanner. The vBMD values were further converted into bone ash density according to the relationship of $\rho_{ash}=0.877\times \rho_{HA}+0.079$ , where $\rho_{ash}$ is the bone ash density, unit in mg/cm^3^ and $\rho_{HA}$ is the HA-equivalent vBMD, unit in mg/cm^3^. Heterogeneous FE model (Figure 1B) was generated by converting the bone ash density to the elastic modulus for each bone element using the density-modulus relationship previously published. Poisson’s ratio for the bone elements was set to 0.30. The FE meshes were generated by converting each bone pixel into 2D 4-node plane stress element (PLANE182). The elastic modulus calculated at each image pixel was mapped to the FE mesh using an in-house developed MATLAB (R2017a, MathWorks, Natick, Massachusetts, United States) code. It should be noted that to ensure the connectivity of bone tissues in the FE analysis, the 2D FE bone models were generated from the processed 2D images, each of which was created by keeping the maximal grayscale values in the pixels calculated from 15 2D images randomly selected from the original 2D image datasets. The 2D images and the corresponding 2D FE models were derived from the transverse plane of the human vertebral plane.

**FIGURE 2:**
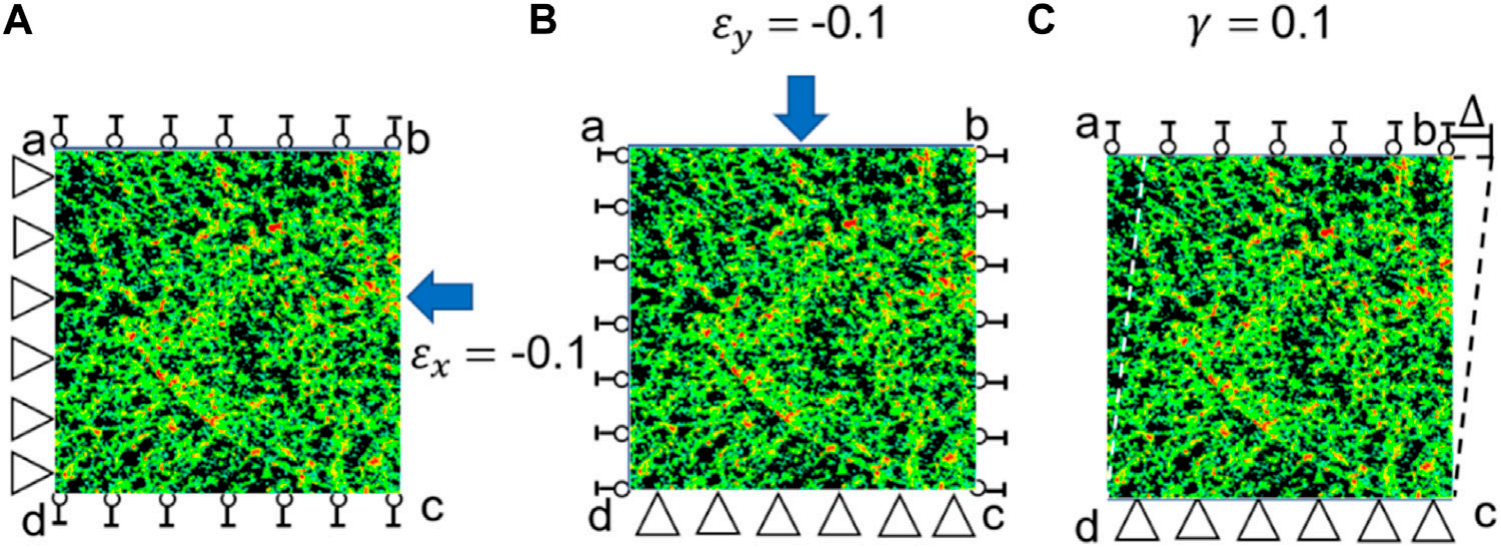
Three loading scenarios used for calculating the elasticity matrix of bone tissue. **(A)** Uniaxial compression in the *x* direction ( $\varepsilon_{x}$ = −0.1), **(B)** uniaxial compression in the *y* axis ( $\varepsilon_{y}$ = −0.1) and **(C)** shear loading in the *x-y* plane ( $\gamma_{xy}$ = 0.1).

The plane stress problem is assumed and the following constitutive model is used to describe the anisotropic mechanical behavior of the bone tissue (Xiao et al., 2021):

$$\left[\begin{array}{c} \sigma_{x}\\ \sigma_{y}\\ \tau_{xy} \end{array}\right]=\left[\begin{array}{ccc} c_{11} & c_{12} & c_{13}\\ c_{12} & c_{22} & c_{23}\\ c_{13} & c_{23} & c_{33} \end{array}\right]\left[\begin{array}{c} \varepsilon_{x}\\ \varepsilon_{y}\\ \gamma_{xy} \end{array}\right]$$ (1)

where $\sigma_{x}$ and $\sigma_{y}$ are the normal stresses in the *x* and *y* directions, respectively, $\tau_{xy}$ is the shear stress in the *x*-*y* plane, $\varepsilon_{x}$ and $\varepsilon_{y}$ are the normal strains in the *x* and *y* directions, respectively, $\gamma_{xy}$ is the shear strain in the *x*-*y* plane and $c_{11},c_{12},\ldots ,c_{33}$ are the elastic constants. Because of the symmetric property of the elasticity matrix, there are six independent constants in the constitutive model, which can be determined using the following three loading scenarios (the FE model was solved three times for the following three loading scenarios to obtain the elastic constants) (Figure 2).

First, the strain in the *x* direction is set to −0.1 while the other two strains are set to zeros, i.e., $\varepsilon_{x}$ = −0.1, $\varepsilon_{y}$ = 0.0, $\gamma_{xy}$ = 0.0 (Figure 2A). Under this loading scenario, the elastic constants of $c_{11}$ , $c_{12}$ and $c_{13}$ can be calculated as below:

$$c_{11}=\frac{\sigma_{x}}{\varepsilon_{x}},c_{12}=\frac{\sigma_{y}}{\varepsilon_{x}},c_{13}=\tau_{xy}/\varepsilon_{x}$$ (2)

Second, the strain in the *y* direction is set to −0.1 and other two strains are set to zeros, i.e., $\varepsilon_{x}$ = 0.0, $\varepsilon_{y}$ = −0.1, $\gamma_{xy}$ = 0.0 (Figure 2B). Under this loading scenario, the elastic constants of $c_{22}$ and $c_{23}$ can be calculated as:

$$c_{22}=\frac{\sigma_{y}}{\varepsilon_{y}},c_{23}=\tau_{xy}/\varepsilon_{y}$$ (3)

Third, the shear strain is set to 0.1 and other two strains are set to zeros, i.e., $\varepsilon_{x}$ = 0.0, $\varepsilon_{y}$ = 0.0, $\gamma_{xy}$ = 0.1 (Figure 2C). Under this loading scenario, the elastic constants of $c_{33}$ can be calculated as:

$$c_{33}=\tau_{xy}/\gamma_{xy}$$ (4)

**FIGURE 3:**
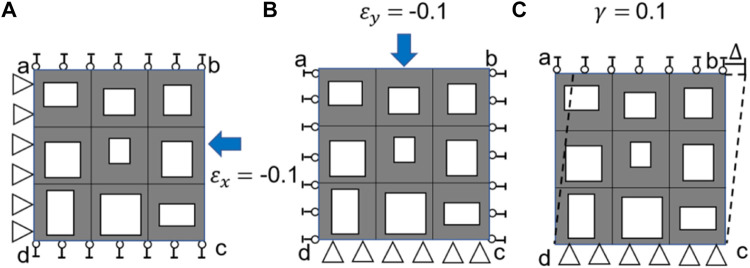
Three loading scenarios used for calculating the elasticity matrix of bone scaffold, **(A)** uniaxial loading in the x direction, **(B)** uniaxial loading in the y direction and **(C)** shear loading in the x-y plane.

It should be noted that the same loading scenarios (Figure 3) and procedure described above was used to calculate the elasticity matrix of the bone scaffold (also meshed using PLANE182) in the subsequent analysis.

### 2.2 Design setting for the bone scaffold

**FIGURE 4:**
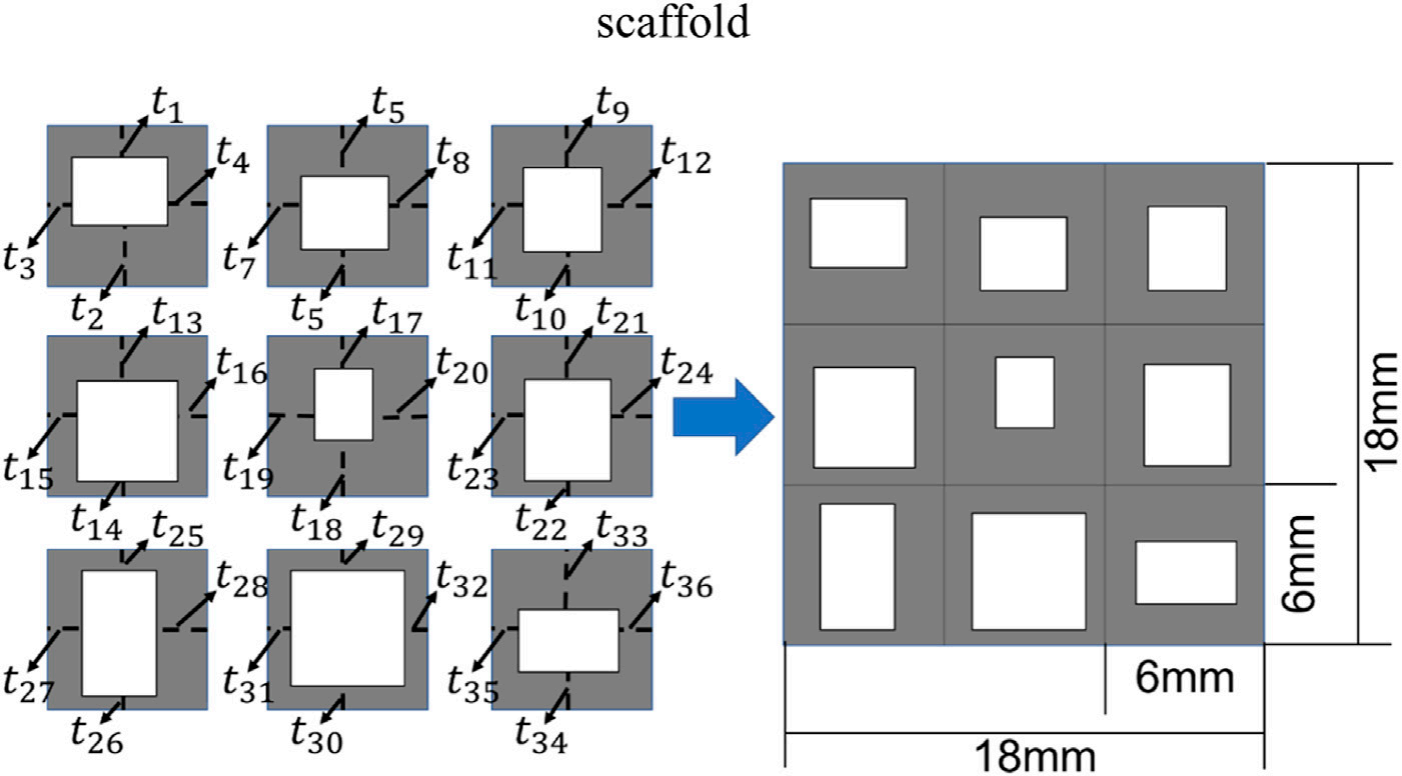
The scheme for designing the bone scaffold using the convolutional neural network (CNN) model (36 independent design variables).

**FIGURE 5:**
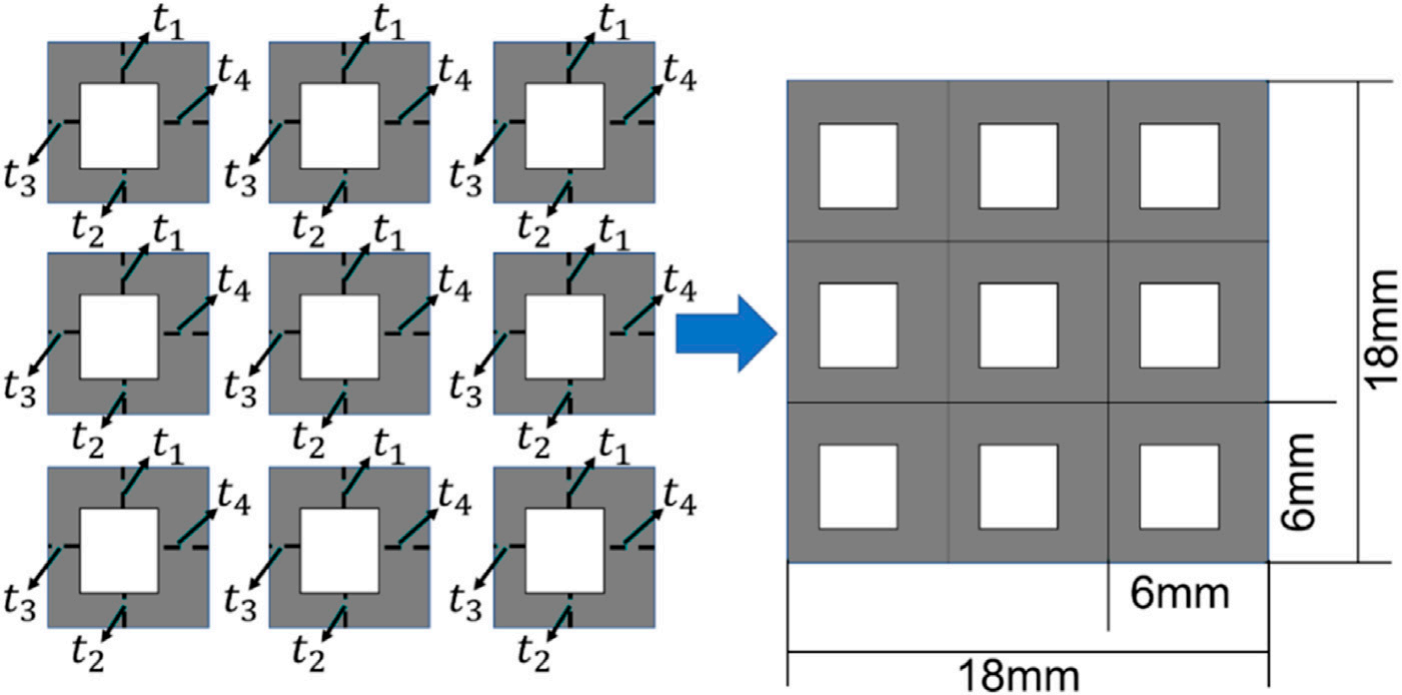
The scheme for designing the bone scaffold using the conventional framework (periodic cell and four independent design variables in each cell).

In the present study, the 2D bone scaffold with the anisotropic mechanical properties was intended to be designed. The porous bone scaffold with 3 × 3 cells was used for the demonstration (Figure 4). Nevertheless, the readers can also use the same framework to design the scaffolds with more cells. The dimension of the scaffold was set to 18.0 × 18.0 mm^2^, which is the size similar to the defected human vertebral part. In each cell of the scaffold, the four dimensional parameters, i.e., the four thicknesses, were set as the independent design variables. Therefore, there are 36 independent design variables ( $t_{1}∼t_{36}$ ) for the entire scaffold. It is obvious that it is very time consuming to perform the structural optimization involving 36 independent design variables using the conventional optimization methods, such as the solid isotropic material with penalization (SIMP) method and the level set method (LSM) (Davoodi et al., 2021). In order to demonstrate the advantage of using 36 independent design variables in the scaffold design, the design using the conventional design framework, i.e., using the periodic cell and four design variables in each cell, was also performed for the comparison purpose (Figure 5).

Considering the minimal structural thickness, which can be produced by the additive manufacturing (e.g., the selective laser melting), is approximately 0.2 mm, the minimal dimension of the design variable was set to 0.2 mm and the thickness of the scaffold was increased or decreased by 0.2 mm in the iterations. The scaffold designed was intended to replace the defected bone tissues. Therefore, to make the scaffold clinically relevant and to increase the computational efficiency, three discrete values, i.e., 0.2, 0.4 and 0.6 mm, were set as the design ranges for both the 36 design variables in the CNN method and the four design variables in the conventional design framework. Because of the constraint on the structural dimensions, the porosity of the scaffold is consequently constrained.

Regarding the characterization of the mechanical property of the bone scaffold, because all the dimensional parameters were set as the independent design, the scaffold designed can exhibit the anisotropic mechanical behavior and thus the constitutive relation presented Eq. 1 was used to describe the mechanical properties of scaffold. In the present study, the Ti-6Al-4V was considered as the base material for producing the porous scaffold. Therefore, in the FE models of 2D bone scaffolds, the Young’s modulus of the solid part was set to 113.8 GPa and the Poisson’s ratio was set to 0.34 (Niinomi, 1998).

In the design of bone scaffolds, the differences in the six elastic constants calculated from the bone and scaffolds may be very large. The constant with a large difference will make a significantly large contribution to the design objective function, leading to the ignorance of the constants with small differences. It is revealed in the authors’ previous study that the contributions of the elastic constants $c_{13}$ and $c_{23}$ to the anisotropic properties of the scaffold are ignorable (Lu et al., 2019b; Li et al., 2019). Therefore, to make the influencing role of each constant approximately the same in the optimization process, the following weighting factors were introduced:

$$w_{ij}=\left[\begin{array}{ccc} \frac{1}{1-\nu^{2}} & \nu \cdot \frac{1}{1-\nu^{2}} & 0\\ \nu \cdot \frac{1}{1-\nu^{2}} & \frac{1}{1-\nu^{2}} & 0\\ 0 & 0 & \frac{1}{2\left(1+\nu \right)} \end{array}\right]$$ (5)

where ν is the Poisson’s ratio of the base material.

The relative differences between the elastic constants of the bone scaffold and those of the bone tissue were calculated using the following formula:

$$a_{ij}={\left(\frac{c_{ij}^{\prime }-c_{ij}}{c_{ij}}\right)}^{2}\;\left(i,j=1,2,3\right)$$ (6)

where $c_{ij}^{\prime }$ are the elastic constants of the bone scaffold and $c_{ij}$ are the elastic constants of the bone tissue. In the equation above, a power of two is used to magnify the difference between different samples. Additionally, it is used to convert the negative values to positive values so as to increase the prediction accuracy.

Then the objective function used in the optimization process was defined as:

$$f=\sum \frac{w_{ij}}{\hat{w}}a_{ij}\;\left(i,j=1,2,3\right)$$ (7)

where $\hat{w}{=}_{\sum w_{ij}}$ .

### 2.3 Machine learning based framework for the optimization of bone scaffold

**FIGURE 6:**
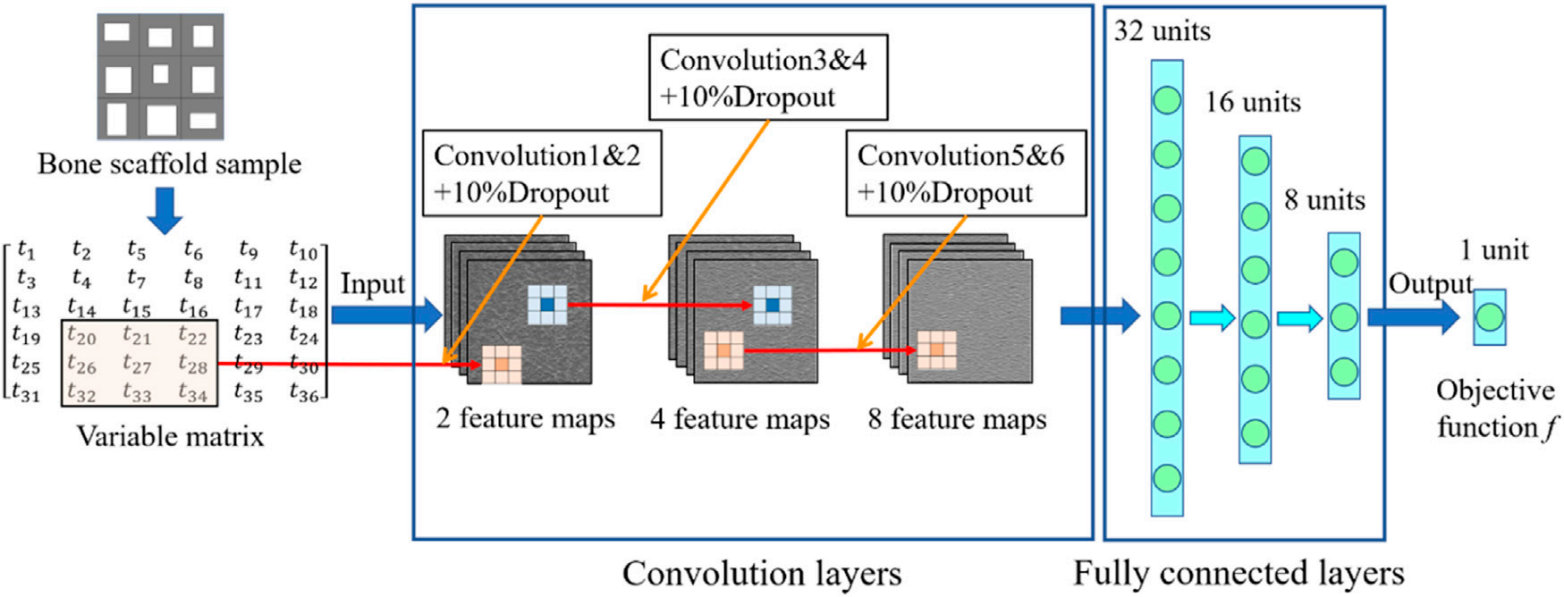
The CNN model developed in the present study.

**FIGURE 7:**
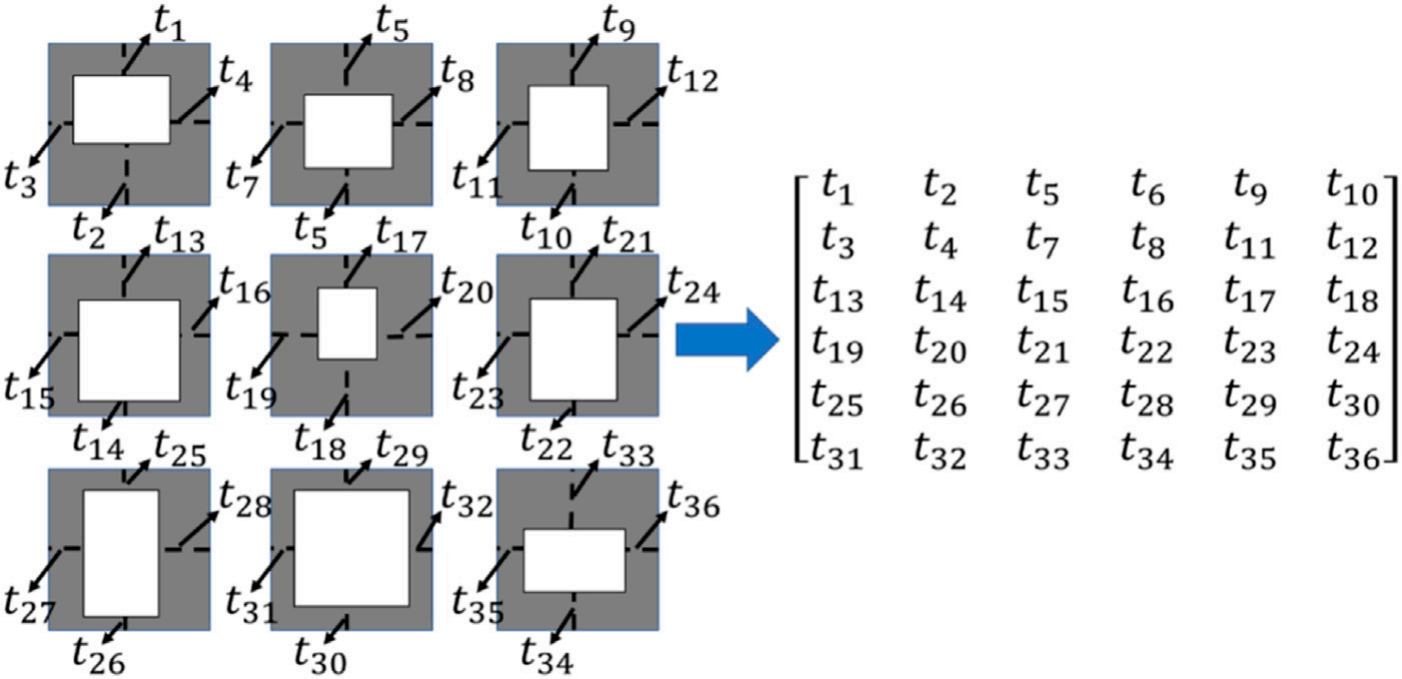
Conversion of the scaffold design into the matrix expressed in Matlab.

In the design of anisotropic bone scaffold, there are 36 independent design variables and each variable has three values to be chosen from. Therefore, there are $3^{36}$ possible designs. It will take a long time to perform the optimization using the FE simulations and the calculation time will be exponentially increased when the number of design variable is increased. To increase the calculation efficiency and make the design clinically practical, a novel self-learning convolutional neural network (CNN) based optimization framework was developed to design the scaffolds. The CNN model constructed is shown in Figure 6, where three convolutional layers and three full-connection layers were used in the model. The convolution kernels were trained in a hierarchical manner, which consisted of low-level features to generate more complex patterns. All convolution kernels were set to a size of 3 × 3. The maximal pooling was applied after the convolutional layers to simplify the information of the output neurons (Li et al., 2019). To improve the accuracy of the CNN model, a dropout rate of 10% was used in the three pooling layers. Each new design of scaffold was converted into the 6 × 6 matrix expressed in Matlab (Figure 7) and serve as the input for the CNN model. The conversion of the design into Matlab matrix enabled the automation of the entire optimization process and increased the calculation process. The output of the CNN is the objective function given in Eq. 8. It should be noted that the CNN architecture presented in the present study is not new but is guided by the work done by Li et al. (2019), which was created to solve a similar problem. Different CNN architectures or machine learning techniques may also be used to achieve the same purpose, i.e., designing anisotropic bone scaffolds. The CNN model presented in the present paper can take the image (in terms of the matrix) as the input and is just one demonstration of the technique in designing anisotropic porous bone scaffolds. The reason that the convolutional layers are used in the CNN architecture is that the computational efficiency can be largely increased, because the convolutional layer can deal with the two-dimensional input while the fully connected layer can only deal with the one-dimensional input.

**FIGURE 8:**
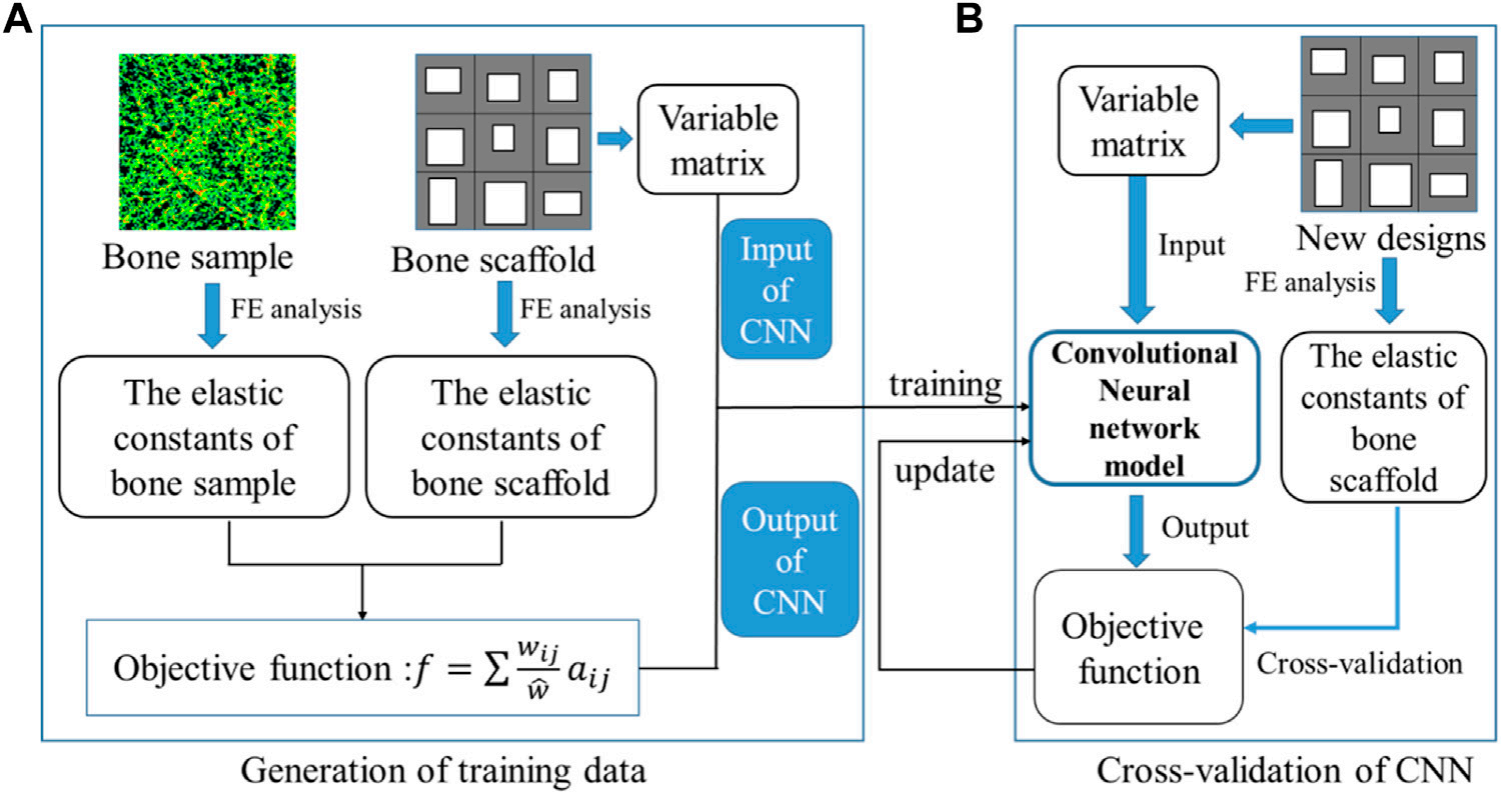
Training and cross-validation of the convolutional neural network model for predicting the elastic constants of bone scaffold, **(A)** generation of the training data and **(B)** cross-validation of the CNN model.

The training and cross-validation of the CNN model is shown in Figure 8. In the training of the CNN model, first, 10,000 bone scaffolds were randomly generated, 8,000 of which were used for the training of the CNN model (Figure 8A) and the remaining 2,000 were used for the cross-validation of the CNN model (Figure 8B). The elastic constants of bone scaffolds calculated from the FE analysis were served as the ground truths for the training and cross-validation. In the training process, the CNN model learned a valid representation describing the geometric features of the bone scaffolds. A loss function was defined to quantify the differences between the elastic constants predicted from the CNN model and those calculated from the FE analysis. The kernels and biases in the convolutional layers and weights in the fully connected layers were then adjusted using the backpropagation algorithm (Rubio et al., 2011). Iterative adjustments were made to minimize the loss function using a large datasets of bone scaffolds. In the present study, the mean absolute error (MAE) between the FE prediction and the CNN prediction was set as the objective (loss) function:

$$MAE\left[Y,f\left(X\right)\right]=\frac{1}{n}\overset{n}{\underset{i=1}{\sum }}\left|Y_{i}-f_{i}\left(X\right)\right|$$ (8)

where $Y_{i}$ is the objective function presented in Eq. 7 which was calculated using the FE method, $f_{i}\left(X\right)$ is the corresponding value calculated using the CNN method and *n* is the number of the samples used for the cross-validation of the CNN model (*n* = 2,000).

To assess the predictive power of the CNN model constructed, 500 new bone scaffolds were processed. The values of these bone scaffolds, calculated as the objective function for optimization, i.e., that presented in Eq. 8, were calculated using the trained CNN model and the FE method, respectively. The FE predictions were served as the ground truths and the predictive power of the CNN model was obtained by comparing the values obtained from the CNN and the FE models. The linear correlation analysis was performed between the CNN and FE predictions using the 500 data samples. Because the variance between the CNN and FE predictions increased with the amplitude of the values, the log transformed values were plotted and analyzed, in which the following transformation formula was used:

$$\mathrm{Log}\_f=\left(\log\left(f\right)+1\right)/\log\left(f_{\max}\right)$$ (9)

where f is the objective function, calculated using Eq. 7, before the log transformation, Log_*f* is the corresponding value after the log transformation and $f_{\max}$ is the maximal value in the optimal sample dataset.

**FIGURE 9:**
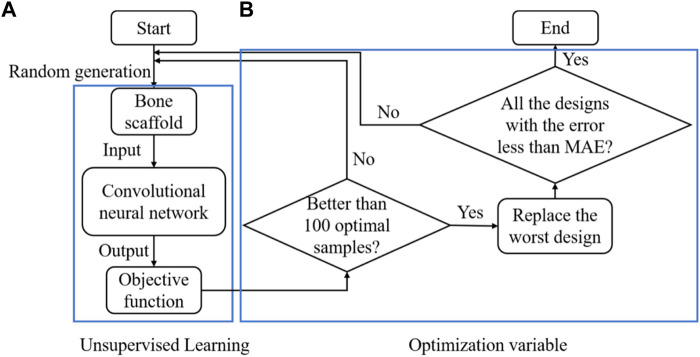
The design of the anisotropic scaffold using the self-learning CNN model, **(A)** unsupervised learning and **(B)** optimization variable.

In summary, the CNN-based framework for designing the anisotropic scaffold is shown in Figure 9. It should be noted that a self-learning process was introduced into the scaffold design to accelerate the optimization process. The self-learning process consists of two parts: unsupervised learning (Figure 9A) and optimization variable (Figure 9B). Specifically, the process can be briefly explained as below: in the iterations, 10,000 new samples were generated from which the first 100 optimal designs were selected as the initial optimal samples. The process was repeated and the first 100 optimal designs were updated after each iteration, i.e., if the design is better than those in the 100 optimal designs and then it is used to replace the worst design in the 100 designs. Because some errors are presented in the CNN model and the MAE is the average prediction error between the prediction and the actual values, to avoid the exclusion of the optimal samples, all the designs with the error less than MAE remained in the iterations.

Using the framework presented in Figure 9, the bone scaffold was designed to mimic the anisotropic mechanical behavior of a specific defected bone sample, which is one of the main design objectives in scaffold design (Gómez et al., 2016; Kang et al., 2020). To demonstrate the ability of the CNN based framework in designing the scaffolds for different bone samples and also to take into account the variances among different bones, 12 bone samples with different porosities and structures, obtained from the HR-pQCT images, were processed and the corresponding scaffolds were designed using the CNN and conventional methods. Statistical analysis was performed and the normality check for all the data samples was performed using the statistical program PASW statistics (SPSS Inc., Chicago, IL) and the probability of type I error was set to *α* = 0.05, i.e., *p* < 0.05 was considered normal distribution.

To quantify the differences between the mechanical properties of the bone scaffolds and those of the bone samples, the relative error (RE) was used, which is defined as below:

$$RE=\frac{P^{CNN}-P^{RVE}}{P^{RVE}}\times 100\%$$ (10)

where $P^{RVE}$ is the elastic constant calculated from the bone samples, $P^{CNN}$ is the corresponding elastic constant calculated from the bone scaffold designed using the self-learning CNN or conventional method.

To enable the process of a large amount of bone scaffolds, all the pre-processing and post-processing were automated using the in-house developed Matlab (R2019, MathWorks, Natick, Massachusetts, United States) code and the FE analysis was performed using the Ansys (v18.0, ANSYS, Inc., Canonsburg, PA, United States). The CNN model was constructed using the Tensorflow 2.0 module in Python 3.7. The training process was conducted on a desktop computer setting to i7-8700 CPU, 32G RAM, and the Nvidia GTX1060. The batch size was set to 128 and the training was iterated for 200 epochs. The training process took approximately 2.0 h.

## 3 Results

### 3.1 Cross-validation and prediction power of the convolutional neural network model

**FIGURE 10:**
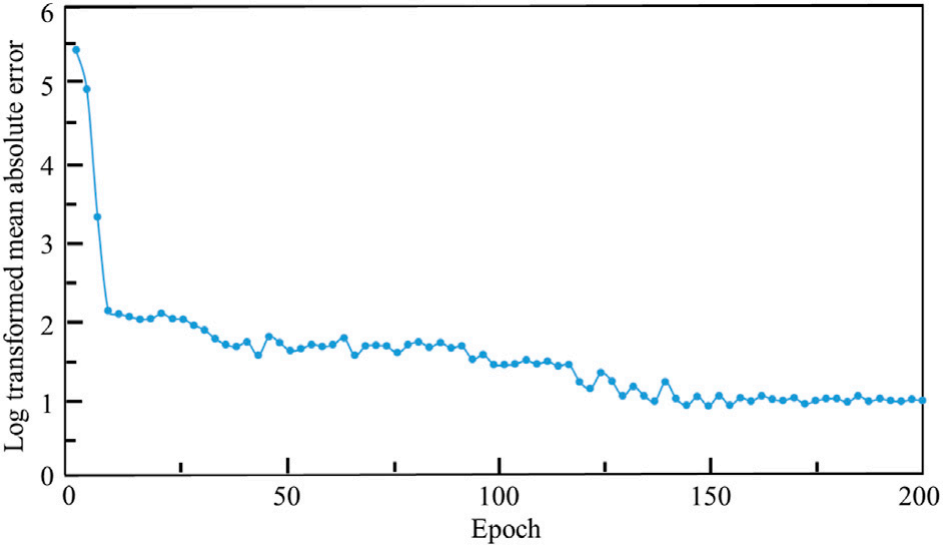
The relationship between the log transformed mean absolute error, where Y = ln (MAE)+1 is used, and the epoch.

**FIGURE 11:**
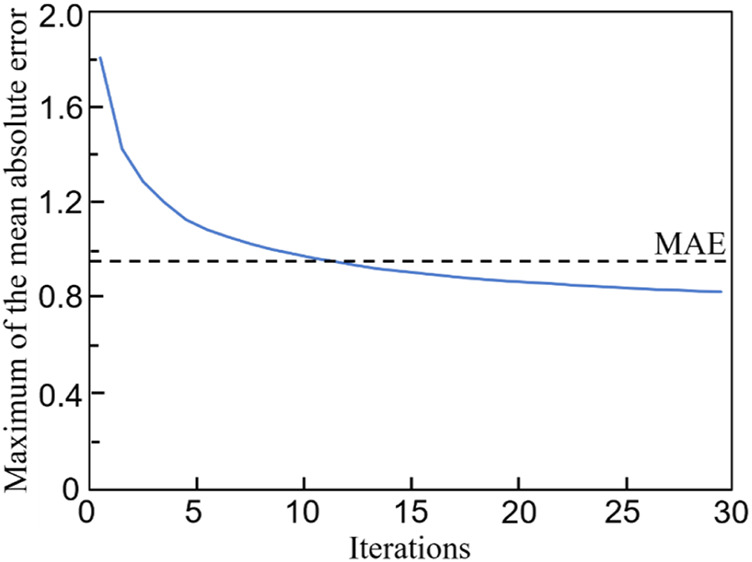
The evolution of the maximum mean absolute error (MAE) in 100 optimal samples with the number of iterations.

The relationship between the mean absolute error (MAE) and the training iterations is shown in Figure 10. Because the initial values of the weights and biases are randomly assigned, the MAEs of the first a few iterations are high. However, after several iterations, the MAE descends rapidly and the MAE is below 1.0 after 120 training epochs. Therefore, no over-fitting is observed in the cross-validation of the CNN model developed. In the process of self-learning accelerated optimization, the changes of samples were recorded. It can be seen that this procedure ensured the errors in all the 100 optimal designs are less than MAE after some iterations (Figure 11). Afterwards, an optimal sample can be obtained from these 100 samples.

**FIGURE 12:**
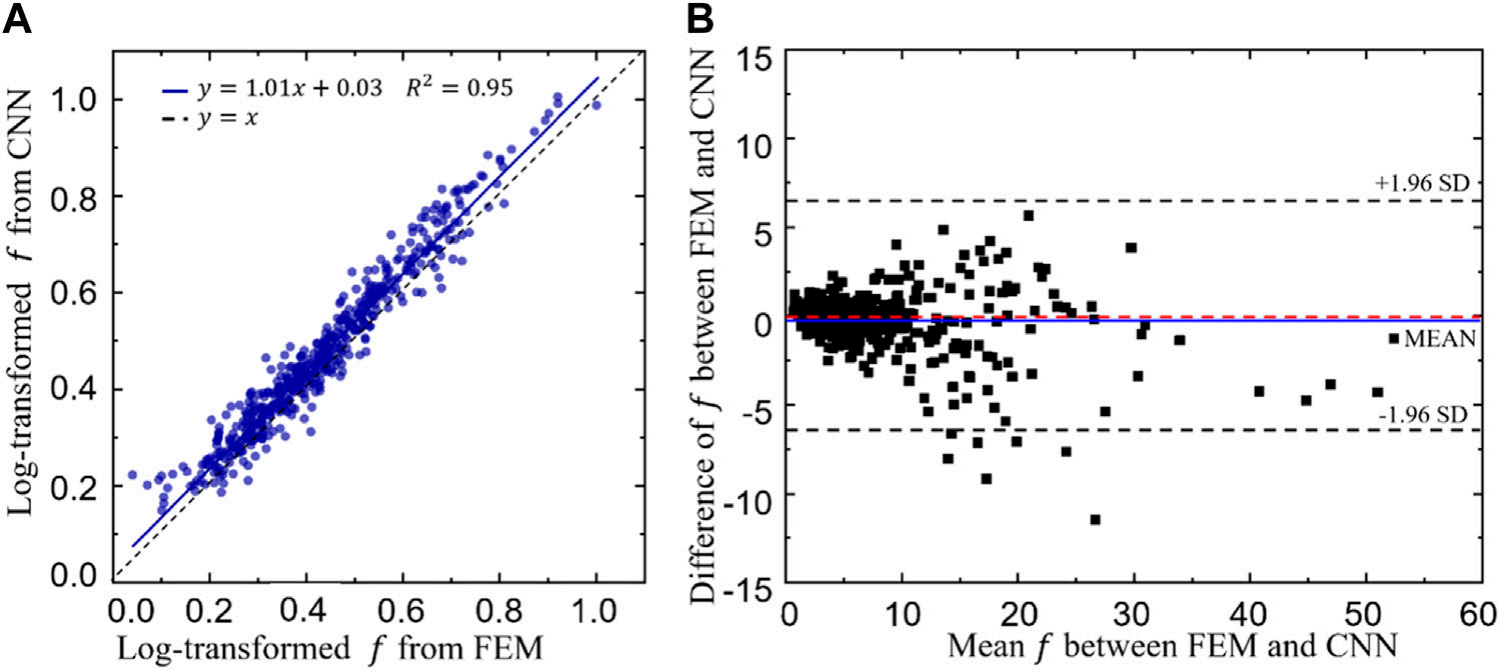
**(A)**The relationship between the log transformed objective functions predicted from the convolutional neural network (CNN) model and those calculated from the finite element method (FEM). **(B)** Bland-Altman diagram of the objective function (*f*) between the FEM and CNN predictions.

The linear correlation between the log transformed objective functions obtained from the CNN and FEM is shown in Figure 12A. A high coefficient of determination (*R* ^2^) has been achieved, i.e. *R* ^2^ = 0.95, implying a good prediction power of the CNN model. To further demonstrate the prediction power of the CNN model, Bland-Altman diagram of the objective function (*f*) between the FEM and CNN predictions is presented in Figure 12B, where the values in the *x* axis represent the mean objective functions obtained from the CNN and FEM predictions (both calculated using Eq. 8 and the values in the *y* axis represent the difference between the objective functions. It is shown in the figure that the mean difference, represented in the blue line, is very close to zero. Additionally, 493 out of 500 samples (98.6%) fall in the confidence interval from −1.96 standard deviation (SD) to +1.96 SD (corresponding to 6.28) and only seven samples are out of this interval, implying a high degree of agreement between the CNN and the FEM predictions.

### 3.2 Design results from the self-learning convolutional neural network model

**FIGURE 13:**
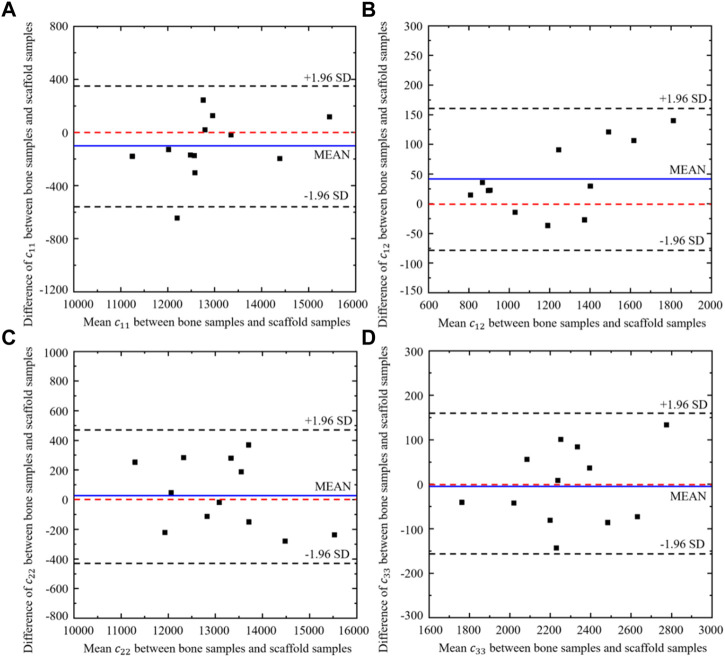
Bland-Altman diagram of elastic constants between the scaffold designed from CNN model and the corresponding bone samples (*n* = 12).

Twelve different bone samples are selected as the tissues to be replaced and the corresponding 12 bone scaffolds are designed using the self-learning CNN model developed. To assess the performance of the self-learning CNN model, the agreement between the elastic constants of the bone samples and those of the designed scaffolds was assessed using the Bland-Altman diagram (Figure 13), where the values in the *x* axis represent the mean elastic constants obtained from the bone samples and the bone scaffolds and the values in the *y* axis represent the differences between the elastic constants of the bone samples and those of bone scaffolds. It is shown in the figure that for the constants $c_{22}$ and $c_{33}$ , the mean differences are very close to zero. The mean differences are −94.0 and 40.5 for the constants $c_{11}$ and $c_{12}$ , respectively, which are also close to zero. The 95% confidence intervals for $c_{11}$ , $c_{12}$ , $c_{22}$ and $c_{33}$ are [−565, 377], [−76, 157], [−427, 482] and [−156, 161], respectively. 91.7% samples of $c_{11}$ and all the $c_{12}$ , $c_{22}$ and $c_{33}$ samples fall in the 95% confidence intervals, implying a high degree of agreement in these constants between the bone samples and the bone scaffolds.

**FIGURE 14:**
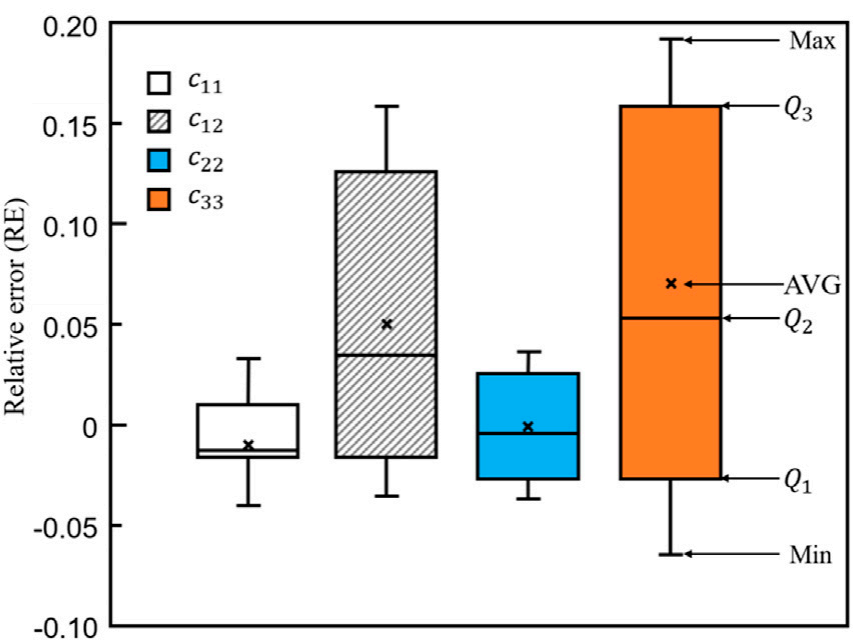
The relative errors in the four elastic constants designed by the self-learning CNN model.

To further demonstrate the differences between bone scaffolds and bone samples, the relative errors in the four elastic constants, calculated using Eq. 10, are presented in Figure 14, where MAX and MIN represent the maximum and minimum values in the dataset, respectively. $Q_{1}$ , $Q_{2}$ and $Q_{3}$ represent the lower quartile, the median and the upper quartile values in the dataset and AVG represents the average value in the dataset. It is shown in the figure that the average errors are −0.01, 0.03, 0.01 and 0.05 for $c_{11}$ , $c_{12}$ , $c_{22}$ and $c_{33}$ , respectively. The ranges from $Q_{1}$ to $Q_{3}$ are much smaller for $c_{11}$ and $c_{22}$ ([−0.02, 0.01] and [−0.03, 0.03], respectively) than those for $c_{12}$ and $c_{33}$ ([−0.02, 0.13] and [−0.03, 0.16], respectively), implying $c_{11}$ and $c_{22}$ can be better matched to those of the native bone tissue using the self-learning CNN model.

### 3.3 Comparison of the results obtained from the convolutional neural network and conventional methods

**FIGURE 15:**
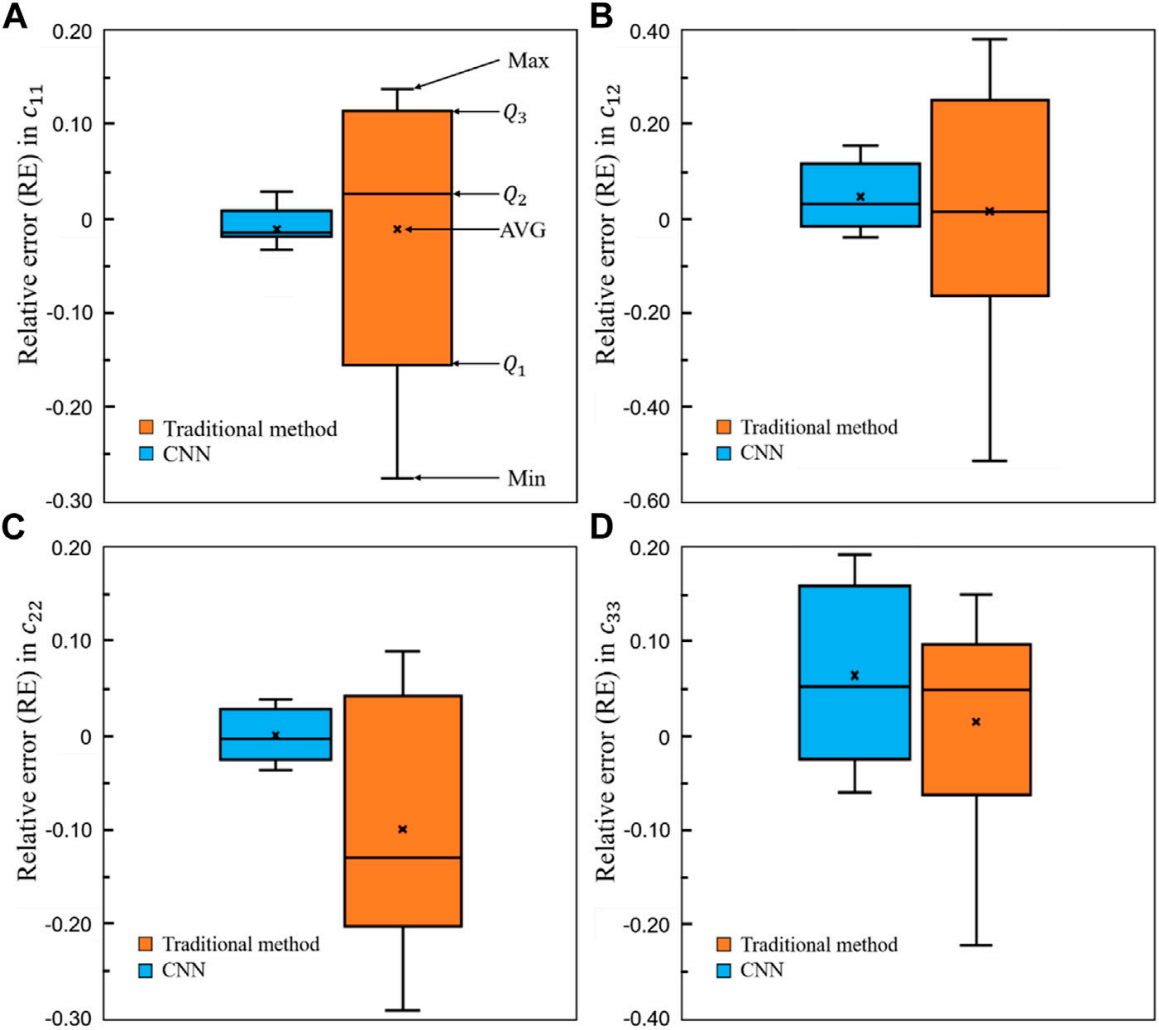
Comparison of the relative errors in the four elastic constants between the self-learning CNN and conventional methods (*n* = 12).

**FIGURE 16:**
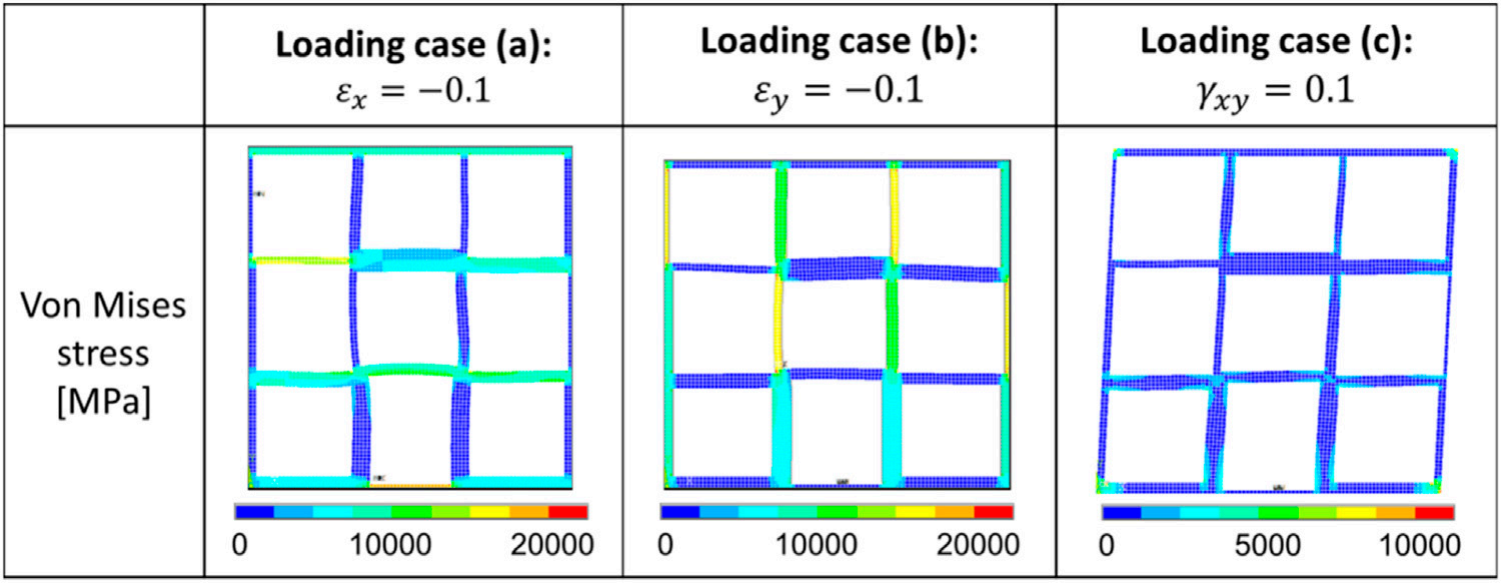
Distribution of the von Mises stress in one representative optimal scaffold under the three different loading cases.

To demonstrate the superior performance of the self-learning CNN model over the conventional method, the relative errors in the four elastic constants are presented in Figure 15. It is shown in the figure that for all the four constants, the average errors are closer to zero in the CNN group than those in the conventional method group. Furthermore, the relative errors from $Q_{1}$ to $Q_{3}$ are much smaller in the CNN group than those in the conventional method group, implying the relative errors are more scattered distributed in the scaffold designed using the conventional method. Figure 15 revealed that using the self-learning CNN model developed, not only a larger range of bone scaffolds can be designed, but also the mechanical properties of bone scaffolds designed from the CNN model are closer to those of the targeted bone samples. Distribution of the von Mises stress in one representative optimal scaffold designed from the CNN model is shown in Figure 16, where variable thicknesses are present in the structure.

## 4 Discussion

In the present study, a novel self-learning convolutional neural network (CNN) model was developed and its performance in designing the bone scaffolds with the anisotropic mechanical properties matched to the targeted bone tissue was demonstrated. The novelty of the present study lies in the novel design of anisotropic bone scaffolds using the emerging self-learning CNN technique.

The present study was motivated by the fact that it is crucial to design bone scaffolds with the anisotropic mechanical properties matched to those of the replaced defected bone tissues, but it is very challenging to realize this using the conventional design approaches because of the large amount of design variables involved. The emerging machine learning technique has the potential to solve the challenges. Indeed, it is revealed in the present study that the design and optimization of one anisotropic scaffold took approximately only 20 min excluding the time used in the training and cross-validation of the CNN model, which took approximately 2 days (including pre-processing of the 10,000 bone scaffolds, the model generation, the FE calculation, the post-processing, etc.). In contrast, it would take much more time to complete the design and optimization involving the same number of design variables, i.e., 36, using the conventional method, such as LSM. It should be noted that the more the independent design variables are involved in the design (e.g., in the 3D case), the more obvious efficient the self-learning CNN method is. The other advantage of the CNN technique is that because there is no constraint on the number of design variable, the design space can be largely expanded. As a consequence, new novel material designs can be made possible using the CNN model. Therefore, the CNN method has the great potential to be used as a novel and crucial tool in the design of 3D porous implants.

Regarding the ability of the self-learning CNN model in designing the scaffolds with anisotropic mechanical properties matched to those of the defected bone, a high ability has been achieved in the present study, which was assessed using the elasticity matrix. It should be noted that when evaluating the mechanical properties of bionic bone scaffolds, it is crucial to use the elasticity matrix of the structure, because the porous scaffolds are not isotropic. Nevertheless, in most previous studies (Dang et al., 2018; Zheng et al., 2021), the mechanical properties of scaffolds under just one or two loading scenarios are investigated and consequently their conclusions are limited to certain conditions.

The bionic scaffold with the mechanical properties matched to those of the native bone tissue is crucial in the clinical application, because when the scaffold is implanted in the human body, it has to be working together with the surrounding soft and hard tissues. If the mechanical properties of the implanted scaffold are too high, the stress shielding may occur and eventually affect the life expectance of the scaffold [ (Gómez et al., 2016), (Bloyer et al., 2007)]. If the mechanical properties of the implanted scaffold are too low, the scaffold may fail to afford the external loadings. Additionally, because of the daily activities of human body, all sorts of loading scenarios may occur in the bone (Huang et al., 2017) and consequently the anisotropic mechanical properties should be taken into account in the design of scaffold. Over the thousand years’ evolution, the native bone tissues have been optimized to be the best structures in daily activities. Therefore, the designed artificially bone scaffold should possess the mechanical properties similar to those of the native bone tissues. The present study has advanced the design of bone scaffold towards this goal and it is demonstrated that the bone scaffolds designed using the CNN model indeed possess the anisotropic mechanical properties very similar to those of the native bone tissue.

Some shortcomings in the present study should be noted. First, only one topology (i.e., rectangular) of the scaffold was investigated. The primary aim of the present study was to demonstrate the application of the CNN technique in the design of scaffolds with anisotropic mechanical properties. Nevertheless, in the future, the scaffolds with other topologies, such as sphere shape, etc. should also be investigated. Second, the simplified method (Figures 2, 3) for reconstructing the elastic matrices of bone and anisotropic scaffolds is used in the present study. More rigorous method such as periodic boundary condition should be incorporated in the future to have an accurate representation of the elastic matrix of the fully anisotropic structure. Third, due to the complexities in the design problem, only the 2D examples were presented and the plane stress scenario was assumed. In the current setting, there are 36 independent design variables and $3^{36}$ possible designs. Extending the current framework to the 3D scenario will create the computational “disaster.” The authors of the present study are in the process to solve this technical challenge for 3D case. Nevertheless, the present study is the first towards the application of the self-learning CNN technique in designing bionic scaffolds with the anisotropic mechanical properties. Regarding the assumption of plane stress scenario, it is believed to be reasonable for the purpose of the present study, i.e., demonstrating the feasibility of the CNN model in designing the anisotropic scaffold involving many design variables. Fourth, only the elastic mechanical properties of the scaffolds were investigated. It should be noted that some other mechanical properties, especially the long-term properties of the bone scaffold, such as the fatigue (Huo et al., 2022), are the crucial factors influencing the life performance of the scaffold and thus should also be investigated in the future. Additionally in the clinical application of bone scaffolds, not only the mechanical properties but also other properties, such as the permeability, the cell behavior, should also be taken into account. Therefore, one of the future works in this direction is to investigate the overall performance of the anisotropic scaffold designed by CNN using the permeability test, the cell culture, the animal testing, etc. Last but not the least, the image datasets from the elderly patients (81.3 ± 7.2 year-old) are used. Nevertheless, this is a methodology paper, in which the high resolution images, i.e., HR-pQCT of human vertebrae (not possible in the clinic scenario so far), are used to obtain the accurate representation of the anisotropic mechanical properties of vertebral body. In the future, the 3D analysis using the images with the clinic resolution needs to be performed and tested before the clinical translation of the method.

In conclusion, 2D bone scaffolds with the anisotropic mechanical properties matched to those of the defected native bone tissue were successfully designed using a self-learning convolutional neural network model. It is revealed in the present study that not only the design space of the scaffolds can be expanded using the CNN method, but also the scaffolds designed by the CNN model possess the anisotropic mechanical properties better matched to those of the native bone tissue. Furthermore, the CNN model is efficient, because once the CNN model is well trained, it takes approximately only 20 min to complete the design of bone scaffold involving 36 independent design variables. Therefore, the CNN model developed possesses great potentials in the design of anisotropic bone scaffolds in the clinical setting.

## Data Availability

The original contributions presented in the study are included in the article/supplementary material, further inquiries can be directed to the corresponding authors.

## References

[B1] Al-Ketan O. Rashid K. Al-Rub A. (2019). Multifunctional mechanical metamaterials based on triply periodic minimal surface lattices. Adv. Eng. Mat. 21 (10), 1900524. 10.1002/adem.201900524

[B2] Ataee A. Li Y. Fraser D. Song G. Wen C. (2018). Anisotropic Ti-6Al-4V gyroid scaffolds manufactured by electron beam melting (EBM) for bone implant applications. Mater. Des. 13, 345–354. 10.1016/j.matdes.2017.10.040

[B3] Bloyer D. R. McNaney J. M. Cannon R. M. Saiz E. Tomsia A. P. Ritchie R. O. (2007). Stress-corrosion crack growth of Si-Na-K-Mg-Ca-P-O bioactive glasses in simulated human physiological environment. Biomaterials 28 (33), 4901–4911. 10.1016/j.biomaterials.2007.08.005 17714778 PMC2227951

[B4] Bonatti C. Mohr D. (2019). Smooth-shell metamaterials of cubic symmetry: Anisotropic elasticity, yield strength and specific energy absorption. Acta Mater. 164, 301–321. 10.1016/j.actamat.2018.10.034

[B5] Bucklen B. S. Wettergreen W. A. Yuksel E. Liebschner M. A. K. (2008). Bone-derived CAD library for assembly of scaffolds in computer-aided tissue engineering. Virtual Phys. Prototyp. 3 (1), 13–23. 10.1080/17452750801911352

[B6] Burton W. S. Myers C. A. Jensen A. Hamilton L. Shelburne K. B. Banks S. A. (2021). Automatic tracking of healthy joint kinematics from stereo-radiography sequences. Comput. Biol. Med. 139, 104945. 10.1016/j.compbiomed.2021.104945 34678483

[B7] Chen Z. Xie Y. M. Wu X. Wang Z. Li Q. Zhou S. (2019). On hybrid cellular materials based on triply periodic minimal surfaces with extreme mechanical properties. Mater. Des. 183, 108109. 10.1016/j.matdes.2019.108109

[B8] Dang M. Saunders L. Niu X. Fan Y. Ma P. (2018). Biomimetic delivery of signals for bone tissue engineering. Bone Res. 6 (3), 25–216. 10.1038/s41413-018-0025-8 30181921 PMC6115422

[B9] Davoodi E. Montazerian H. Mirhakimi A. S. Zhianmanesh M. Ibhadode O. Shahabad S. I. (2021). Additively manufactured metallic biomaterials. Bioact. Mater. 15, 214–249. 10.1016/j.bioactmat.2021.12.027 35386359 PMC8941217

[B10] Finkemeier C. G. (2002). Bone-grafting and bone-graft substitutes. J. Bone Jt. Surgery-American Volume 84 (3), 454–464. 10.2106/00004623-200203000-00020 11886919

[B11] Gómez S. Vlad M. D. López J. Fernández E. (2016). Design and properties of 3D scaffolds for bone tissue engineering. Acta Biomater. 42, 341–350. 10.1016/j.actbio.2016.06.032 27370904

[B12] Gu G. X. Chen C. T. Richmond D. J. Buehler M. J. (2018). Bioinspired hierarchical composite design using machine learning: Simulation, additive manufacturing, and experiment. Mat. Horiz. 5 (5), 939–945. 10.1039/C8MH00653A

[B13] Huang K. L. Marcora E. Pimenova A. A. Di-Narzo A. F. Kapoor M. Jin S. C. (2017). A common haplotype lowers PU.1 expression in myeloid cells and delays onset of Alzheimer's disease. Nat. Neurosci. 20 (8), 1052–1061. 10.1038/nn.4587 28628103 PMC5759334

[B14] Huo Y. Lu Y. Meng L. Meng L. Wu J. Gong T. (2021). A critical Review on the design, manufacturing and assessment of the bone scaffold for large bone defects. Front. Bioeng. Biotechnol. 9, 753715. 10.3389/fbioe.2021.753715 34722480 PMC8551667

[B15] Huo Y. Lyu Y. Bosiakov S. Han F. (2022). A critical Review of the design, manufacture, and evaluation of bone joint replacements for bone repair. Materials 15 (1), 153. 10.3390/ma15010153 PMC8746215 35009299

[B16] Kang J. Dong E. Li D. Dong S. Zhang C. Wang L. (2020). Anisotropy characteristics of microstructures for bone substitutes and porous implants with application of additive manufacturing in orthopaedic. Mater. Des. 191, 108608. 10.1016/j.matdes.2020.108608

[B17] Laurencin C. Khan Y. El-Amin S. F. (2006). Bone graft substitutes. Expert Rev. Med. Devices 3 (1), 49–57. 10.1586/17434440.3.1.49 16359252

[B18] Li X. Liu Z. Cui S. Luo C. Li C. Zhuang Z. (2019). Predicting the effective mechanical property of heterogeneous materials by image based modeling and deep learning. Comput. Methods Appl. Mech. Eng. 347, 735–753. 10.1016/j.cma.2019.01.005

[B19] Liu F. Mao Z. Zhang P. Zhang D. Jiang J. Ma Z. (2018). Functionally graded porous scaffolds in multiple patterns: New design method, physical and mechanical properties. Mater. Des. 160, 849–860. 10.1016/j.matdes.2018.09.053

[B20] Lu Y. Krause M. Bishop N. Sellenschloh K. Glüer C. C. Püschel K. (2015). The role of patient-mode high-resolution peripheral quantitative computed tomography indices in the prediction of failure strength of the elderly women's thoracic vertebral body. Osteoporos. Int. 26 (1), 237–244. 10.1007/s00198-014-2846-7 25135580

[B21] Lu Y. Zhu Y. Krause M. Huber G. Li J. (2019a). Evaluation of the capability of the simulated dual energy X-ray absorptiometry-based two-dimensional finite element models for predicting vertebral failure loads. Med. Eng. Phys. 69, 43–49. 10.1016/j.medengphy.2019.05.007 31147202

[B22] Lu Y. Zhao W. Cui Z. Zhu H. Wu C. (2019b). The anisotropic elastic behavior of the widely-used triply-periodic minimal surface based scaffolds. J. Mech. Behav. Biomed. Mat. 99, 56–65. 10.1016/j.jmbbm.2019.07.012 31344523

[B23] Niinomi M. (1998). Mechanical properties of biomedical titanium alloys. Mater. Sci. Eng. A 243 (1), 231–236. 10.1016/S0921-5093(97)00806-X

[B24] Peng X. Huang Q. Zhang Y. Zhang X. Shen T. Shu H. (2021). Elastic response of anisotropic Gyroid cellular structures under compression: Parametric analysis. Mater. Des. 205, 109706. 10.1016/j.matdes.2021.109706

[B25] Rane L. Ding Z. McGregor A. H. (2019). Deep learning for musculoskeletal force prediction. Ann. Biomed. Eng. 47 (3), 778–789. 10.1007/s10439-018-02190-0 30599054 PMC6445355

[B26] Rubio J. J. Angelov P. Pacheco J. (2011). Uniformly stable backpropagation algorithm to train a feedforward neural network. IEEE Trans. Neural Netw. 22 (3), 356–366. 10.1109/TNN.2010.2098481 21193374

[B27] Tan R. K. Zhang N. L. Ye W. (2020). A deep learning–based method for the design of microstructural materials. Struct. Multidiscipl. Optim. 61 (4), 1417–1438. 10.1007/s00158-019-02424-2

[B28] Vijayavenkataraman S. Zhang L. Zhang S. Zhang S. His F. Jerry Y. (2018). Triply periodic minimal surfaces sheet scaffolds for tissue engineering applications: An optimization approach toward biomimetic scaffold design. ACS Appl. Bio Mat. 1 (2), 259–269. 10.1021/acsabm.8b00052 35016376

[B29] Wang G. Shen L. Zhao J. Liang H. Xie D. Tian Z. (2018). Design and compressive behavior of controllable irregular porous scaffolds: Based on voronoi-tessellation and for additive manufacturing. ACS Biomater. Sci. Eng. 4 (2), 719–727. 10.1021/acsbiomaterials.7b00916 33418759

[B30] Xiao P. Haque E. Zhang T. Dong X. N. Huang Y. Wang X. (2021). Can DXA image-based deep learning model predict the anisotropic elastic behavior of trabecular bone? J. Mech. Behav. Biomed. Mater. 124, 104834. 10.1016/j.jmbbm.2021.104834 34544016

[B31] Zheng X. Chen T. T. Guo X. Samitsu S. Watanabe I. (2021). Controllable inverse design of auxetic metamaterials using deep learning. Mater. Des. 211, 110178. 10.1016/j.matdes.2021.110178

